# Association of Covid-19 with blood type A in relation to blood sugar, urea, and blood test (D-dimer and ferritin) in patients from Al-Najaf

**DOI:** 10.25122/jml-2021-0239

**Published:** 2022-02

**Authors:** Hayfaa Jaber Hussein, Sana’a Abdulrazzaq Ibrahim, Shurooq Wesam Al-Shaibani, Noor Hassan Abdulrudha

**Affiliations:** 1. Department of Basic Sciences, Faculty of Dentistry, University of Kufa, AL-Najaf, Iraq

**Keywords:** COVID-19, blood group, blood sugar, blood urea, serum D-dimer, ferritin

## Abstract

COVID-19 is an emerging infectious disease caused by the novel enveloped single-stranded RNA virus quickly declared a pandemic. This study aimed to investigate the severity of COVID-19 infection in patients with blood group type A. A cross-sectional study was conducted at Al-Amal specialized hospital, Al-Najaf (March 8 to March 20/2021). The study included 123 hospitalized patients (63 females and 60 males), aged between 15-95 years, diagnosed with COVID-19, tested for blood group, blood sugar, blood urea, D-dimer, and serum ferritin. Results indicated significant differences in blood sugar and D-dimer in patients with type A blood group at P>0.05. At the same time, no significant difference was found in blood urea and ferritin at P>0.05. The majority of patients showed elevated levels of blood sugar, blood urea, serum D-dimer and ferritin. COVID-19 can infect people of all ages and causes severe infection in all blood groups.

## Introduction

COVID-19 is an emerging infectious disease caused by the novel enveloped single-stranded RNA virus that quickly resulted in an outbreak and was declared a pandemic by the World Health Organization (WHO) [1–3]. It is mainly a respiratory illness highly contagious spread by droplet transmission, causing a spectrum of illnesses from mild sore throat to serious viral pneumonia requiring hospitalization. Furthermore, it may affect multiple organs causing multisystem failure, which disrupts normal immune responses and may cause symptoms including pneumonia, fever, cough, expectoration, and hemoptysis [4–5]. Extrapulmonary damage of COVID-19 involves acute kidney injury, hepatocellular injury, neurological illnesses, myocardial dysfunction and arrhythmia, and gastrointestinal symptoms leading to severe respiratory failure, kidney injury, myocardial injury, and death [6]. Viral load was a considerably predictive marker of severe diseases in older patients [7–8]. A better understanding of the possible risk factors related to disease immunopathology associated with COVID-19 severity is helpful for clinicians in diagnosing patients at high risk and requiring immediate treatment to prevent disease progression and avoid adverse consequences [9–10]. The severity of infection is mainly related to an individual’s immune response, age, and co-morbidities, as well as blood group which was associated with some viruses such as SARS-CoV-1, especially anti-A blood group antibodies, thought to block angiotensin-converting enzyme II (ACE2), which is cell entry receptor for COVID-19 [11–14]. This study aims to correlate the role of different biomarkers in COVID-19 and the severity of the disease in association with blood group type A.

## Material and Methods

A cross-sectional study was conducted at Al-Amal specialized hospital, Al-Najaf (March 8 to June 10/2021). The study included 123 patients (63 female and 60 male) aged between 15–95 years. Patients were already diagnosed with COVID-19 infection using real-time polymerase chain reaction (RT-PCR). All patients were tested for blood group, blood sugar, urea, D-dimer, and serum ferritin by withdrawing 6 ml of peripheral venous blood.

### Procedures

The blood samples were divided into two parts; one part included 5 ml of clotted blood centrifuged for about 15 minutes to get a clear serum for the biochemical tests, and 1 ml from the whole blood was added into the EDTA tube for blood grouping. The blood urea, serum creatinine, serum ferritin, and D-dimer were examined automatically using a Fujifilm fully-automated machine made in Japan. Serum ferritin and D-dimer were determined using Vidas fully-automated machine made in France. Blood grouping for each patient was performed using three drops of whole blood against anti-A, anti-B, and anti-D antigens using the slide method from the Anti ABD kit, which worked manually.

### Statistical analysis

Data analysis was performed using statistical software (Microsoft Excel, version 2016). Data were compared using paired sample T-test, frequency, and cumulative histogram. Statistical significance was defined as P≤0.05; if the P-value was greater than 0.05, the results were regarded as non-significant, but if it was lower than or equal to 0.05, the results were regarded statistically significant.

## Results

During the study period, 123 patients (male and female) infected with COVID-19 were included. Their clinical records were analyzed, including age, biochemical marker, and the ABO blood group distribution. The percentage of blood group A was 67/123 (54.4%), while blood groups B, O, and AB were 33/123 (26.8%), 22/123 (17.8%), and 1/123 (0.8%), respectively. Age ranged from 15–95 years (Table 1).

**Table 1.** Distribution of patient's age regarding blood group.

**Age**	**Blood group A**	**Blood group B, AB, O**
**Mean**	64	58
**Median**	65	60
**Mode**	65	61
**Minimum**	15	18
**Maximum**	95	80

**Figure 1.**
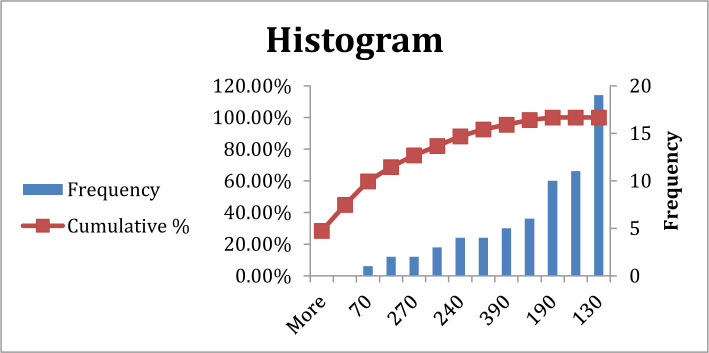
Frequency of blood sugar in type A blood group patients.

**Figure 2.**
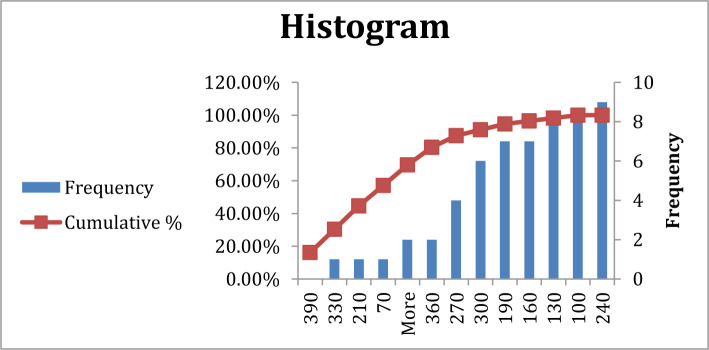
Frequency of blood sugar in group B, AB, and O patients.

There was a significant difference in blood sugar between patients with blood group A compared to other blood groups at P=0.03, which means blood sugar increased in blood group A more than the others, at 130–190 mg/dl level, while other blood groups at 130–240. However, most patients revealed elevated blood sugar levels. (Table 2, Figure 1 and 2).

**Table 2.** Association blood group and blood sugar in patients with COVID-19.

**Blood Sugar**	**Blood group A**		**Blood group B**		**Blood group AB**		**Blood group O**	
	**No.**	**%**	**No.**	**%**	**No.**	**%**	**No.**	**%**
**Normal**	9	13.5	8	24.3	1	100	6	27.3
**Increased**	58	86.5	24	72.7	0	0	16	72.7
**Decreased**	0	0	1	3	0	0	0	0
**Total**	67	100	33	100	1	100	22	100

Normal value: 70–110 mg/dl.

**Figure 3.**
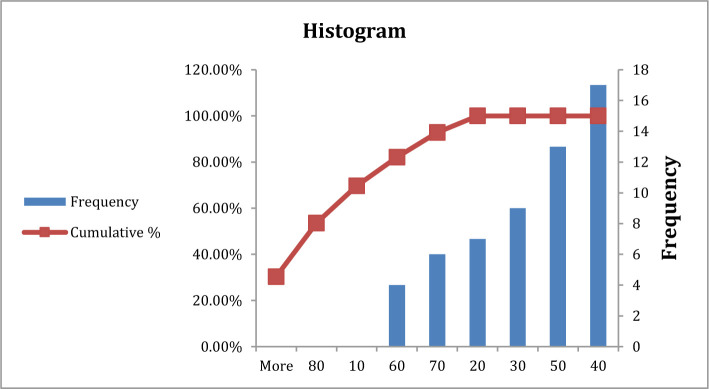
Frequency of blood urea in type A blood group patients.

**Figure 4.**
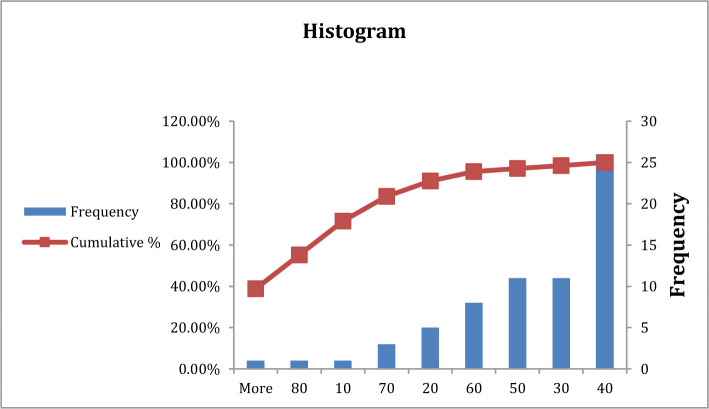
Frequency of blood urea in type B, AB, and O blood group patients.

In the current study, there was no significant difference in blood urea between patients with blood group A in comparison with other blood groups (P>0.05), both groups having the same frequency at (40) mg/dl (Table 3, Figures 3 and 4). Previous studies showed an increased level of blood urea in patients with COVID-19 during infection, which agrees with the current study in which patients have signs of kidney disease that may be a consequence or complication of COVID-19 infection [15–17].

**Table 3.** Association ABO blood group and blood urea in patients with COVID-19.

**Blood urea**	**Blood group A**		**Blood group B**		**Blood group AB**		**Blood group O**	
	**No.**	**%**	**No.**	**%**	**No.**	**%**	**No.**	**%**
**Normal**	9	13.5	5	18.2	0	0	3	13.7
**Increased**	57	85	27	81.8	1	100	19	86.3
**Decreased**	1	1.5	0	0	0	0	0	86.3
**Total**	67	100	33	100	1	100	22	100

Normal value: 8–23 mg/dl.

**Figure 5.**
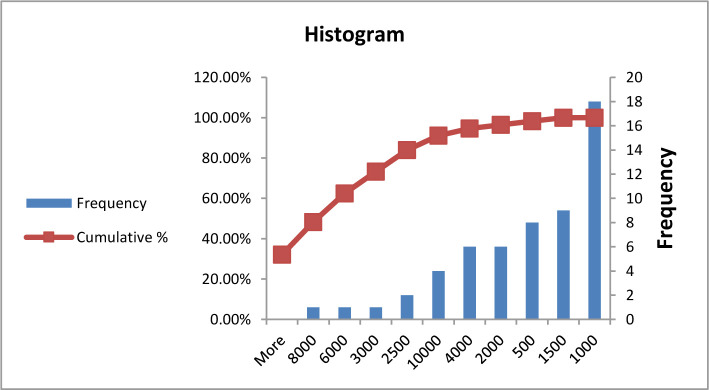
Frequency of D-Dimer in type A blood group patients.

**Figure 6.**
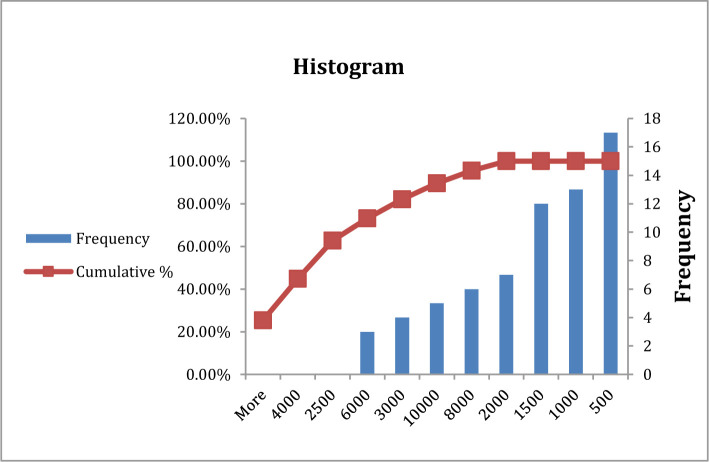
Frequency of D-Dimer in type B, AB, and O blood group patients.

There was a significant difference in D-Dimer between patients with blood group A in comparison with other blood groups at P=0.04, which mean D-Dimer increased in blood group A more than other blood groups, with frequency highly at (500–1000) mg/dl, while blood group A frequency at (1000–1500), more severe infection in blood group A than other blood groups (Table 4, Figures 5 and 6). The current study revealed a notable D-Dimer increase, which may be related to the fact that most patients were aged between 55 and 65, at high risk of developing a complication. These results are consistent with other studies, indicating an increased level of D-Dimer during infection [18].

**Table 4.** Association ABO Blood group and D-dimer in patients with COVID-19.

**D-Dimer**	**Blood group A**		**Blood group B**		**Blood group AB**		**Blood group O**	
	**No.**	**%**	**No.**	**%**	**No.**	**%**	**No.**	**%**
**Normal**	17	41.3	4	15.2	0	0	4	18.2
**Increased**	40	59.7	28	84.8	1	100	18	81.8
**Total**	67	100	33	100	1	100	22	100

Normal value: 0–500 ng/ml.

**Figure 7.**
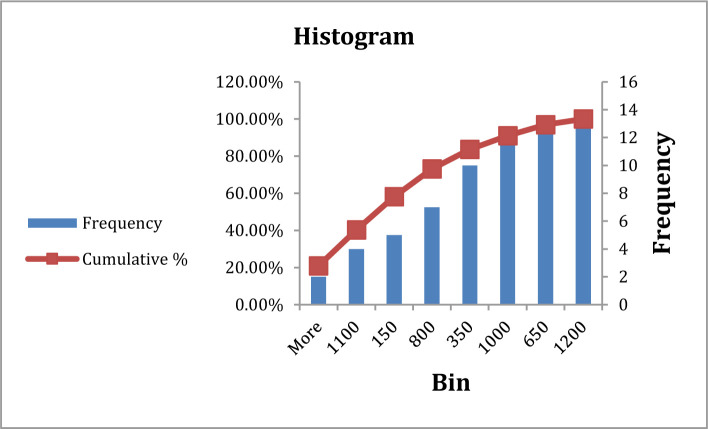
Frequency of ferritin in type A blood group patients.

**Figure 8.**
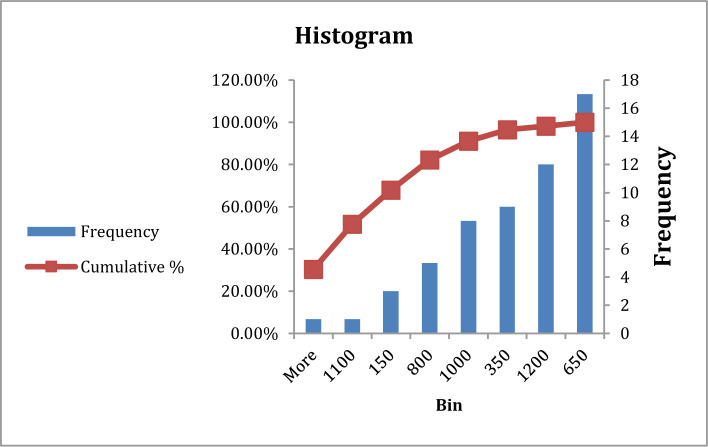
Frequency of ferritin in type B, AB, and O blood group patients.

There was no significant difference in ferritin between patients with blood group A in comparison with other blood groups at P>0.05, with frequency highly at 650–1200 mg/dl for all blood groups, which was considered a sign of severe infection with COVID-19 (Table 5, Figures 7 and 8).

**Table 5.** Association ABO Blood group and ferritin in patients with COVID-19.

**Ferritin**	**Blood group A**		**Blood group B**		**Blood group AB**		**Blood group O**	
	**No.**	**%**	**No.**	**%**	**No.**	**%**	**No.**	**%**
**Normal**	5	7.5	1	3.1	0	0	2	9.1
**Increased**	62	92.5	32	96.9	1	100	20	90.9
**Decreased**	0	0	0	0	0	0	0	0
Total	67	100	33	100	1	100	22	100

Normal value: 13–150 ng/ml.

## Discussion

There was no relation between the blood group and age group or severity of infection in the current study. These may be related to public health status, geographical distribution, and social life, which is compatible with previous studies that showed that people can get infected at any age irrespective of a blood group type. Severe infection can appear at any age, and all included patients were hospitalized. Some studies indicated no relation between blood type ABO and death among hospitalized patients with COVID-19. Also, the mortality rate was the highest in blood group B [11, 19–21]. The differences in results among studies may be related to biological differences and geographical distribution for patients since many reports indicate that blood groups AB, A, and B are at high risk of being infected with COVID-19. In particular, group A was linked to death while group O was at low risk of infection, suggesting that ABO antigens may have a notable role in the pathogenesis of COVID-19 [22].

A study in China showed abnormal fasting blood sugar levels and a highly associated death rate with an increased level of blood sugar. Many studies revealed that stress hyperglycemia could occur in patients with COVID-19 due to an acute blood glucose disorder. Long-term hyperglycemia could induce abnormal coagulation function, endothelial dysfunction, and inflammatory cytokine overproduction caused by abnormal immune activation, so it is important to control blood glucose level during infection, which may be associated with negative consequences or death [23–26].

Elevated D-dimer levels cause impaired coagulation time with fatal results due to disseminated intravascular coagulation (DIC), circulation of thrombin freely, without any control by blood anticoagulants, can make platelets activated and lead to fibrinolysis [27, 28].

Our study revealed that nearly all patients had elevated ferritin levels; these levels agree with other studies indicating increased ferritin levels, indirectly indicated for acute respiratory distress syndrome and severe COVID-19 infection, causing a profuse inflammation cytokine storming, which could cause death [29]. A study in Kirkuk in Iraq [22] included 84 patients (34 female and 50 male) with COVID-19 revealed for most patients with A-type blood group, decreased white blood cells (WBC) count, and increased level of blood urea serum ferritin and D-dimer.

## Conclusion

Infection with COVID-19 may occur in all blood groups with the same degree of severity, but most patients had increased levels of blood sugar, blood urea, D-Dimer, and serum ferritin.

## Acknowledgments

### Conflict of interest

The authors declare no conflict of interest.

### Ethical approval

The study was approved by the ethical committee of the Faculty of Medicine, University of Kufa (1049 KU-8^th^ March 2021).

### Consent to participate

Written informed consent was obtained from all patients.

### Authorship

HJH contributed to data collection. SAI contributed to writing the original draft, methodology, and editing. SWA-S contributed to conceptualizing, and NHA contributed to data analysis.
